# Drugging the unfolded protein response in acute leukemias

**DOI:** 10.1186/s13045-015-0184-7

**Published:** 2015-07-16

**Authors:** Behzad Kharabi Masouleh, Eric Chevet, Jens Panse, Edgar Jost, Michael O’Dwyer, Tim H. Bruemmendorf, Afshin Samali

**Affiliations:** Department of Hematology, Oncology, Hemostaseology and Stem Cell Transplantation, Medical Faculty, RWTH Aachen University, Aachen, Germany; Université Rennes 1 - ER_440 “Oncogenesis, Stress & Signaling”, Centre de Lutte Contre le Cancer Eugène Marquis, Rennes, France; Apoptosis Research Centre (ARC), National University of Ireland, Galway, Ireland; Department of Medicine, National University of Ireland, Galway, Ireland; Department of Biochemistry, National University of Ireland, Galway, Ireland

**Keywords:** Acute myeloid leukemia, Acute lymphoblastic leukemia, Leukemia stem cells, Unfolded protein response, XBP1, Small-molecule inhibitors

## Abstract

The unfolded protein response (UPR), an endoplasmic reticulum (ER) stress-induced signaling cascade, is mediated by three major stress sensors IRE-1α, PERK, and ATF6α. Studies described the UPR as a critical network in selection, adaptation, and survival of cancer cells. While previous reviews focused mainly on solid cancer cells, in this review, we summarize the recent findings focusing on acute leukemias. We take into account the impact of the underlying genetic alterations of acute leukemia cells, the leukemia stem cell pool, and provide an outline on the current genetic, clinical, and therapeutic findings. Furthermore, we shed light on the important oncogene-specific regulation of individual UPR signaling branches and the therapeutic relevance of this information to answer the question if the UPR could be an attractive novel target in acute leukemias.

## Introduction

Although the therapy of acute leukemias either originating from myeloid (acute myeloid leukemia (AML)) or lymphoid lineage (acute lymphoblastic leukemia (ALL)) has improved in recent decades, the heterogeneous genetic landscape of these diseases causes relapse in the majority of patients. The unfolded protein response (UPR) is a conserved adaptive signaling pathway aiming to restore protein homeostasis mainly in the ER. Recent studies suggest an important function in acute leukemias. In this review, we will summarize these results highlighting the druggability of the UPR and give an outlook of potential mechanisms.

## The unfolded protein response

Cell survival is largely dependent on correct production, control, and folding of proteins. To maintain cellular protein homeostasis (proteostasis) and to be shielded against stress stimuli accumulating within the endoplasmic reticulum (ER) causing “ER stress,” cells are mainly dependent on the cytoprotective network of the UPR [1–3]. While under acute conditions, UPR activation acts pro-survival; continuous and chronic stress causes a shift of UPR signaling in acting pro-apoptotic [4, 5]. Thereby, understanding the complex role of the UPR is also understanding not only the specific pathways and molecules both in their time but also cell-specific context [3, 6].

The correct function of the UPR is mediated through three distinct signaling branches in a time- and stimuli-specific manner, namely inositol-requiring enzyme 1 alpha (IRE-1α), PKR-like ER kinase (PERK), and activating transcription factor 6 alpha (ATF6α) [7]. Under unstressed conditions, the stress sensors of PERK, IRE-1α, and ATF6α are maintained inactive through binding to the ER chaperone heat shock 70 kDa protein 5/78 kDa glucose-regulated protein (HSPA5/GRP78). Through various stimuli, such as accumulation of misfolded proteins within the ER, GRP78 binds with a higher affinity to their exposed hydrophobic domains, dissociating from UPR sensors, thereby priming IRE-1α and PERK for oligomerization and autotransphosphorylation [8] and revealing an ER export motif in ATF6α [7].

### PERK-eIF2α

Upon activation, PERK phosphorylates eukaryotic translation initiation factor 2 subunit alpha translation initiation factor (eIF2α) at serine 51 (S51) [9], thereby attenuating global protein synthesis to reduce the number of proteins entering ER, imparting a pro-survival effect on the cells. This prevents assembly of the 80S ribosome translation initiation complex and allows selective expression of activating transcription factor 4 (ATF4). ATF4 then enters the nucleus activating ER stress-response genes involved in protein folding, antioxidant responses, autophagy, amino acid metabolism, and apoptosis promoting cell survival [10–13]. Moreover, PERK activation leads to nuclear factor (erythroid-derived 2)-like 2 (NRF2) phosphorylation and subsequent control of the antioxidant pathway [14].

### ATF6α

 The second branch is represented by the basic leucine zipper transcription factor ATF6α. ATF6α activation occurs in the Golgi complex following export from the ER upon ER stress [15]. ATF6α is then cleaved on both sides of the membrane by site-1 (S1P, also named membrane-bound transcription factor peptidase, site 1 MBTPS1) and site-2 proteases (S2P, also named membrane-bound transcription factor peptidase, site 2 MBTPS2) generating an active transcription factor through regulated intramembrane proteolysis [16]. Following its cleavage, the ATF6α cytosolic domain translocates to the nucleus and activates specific transcriptional programs that promote adaptation, including upregulation of various components of ER-associated degradation (ERAD) [17].

### IRE-1α—XBP1/RIDD

 The most evolutionary conserved arm of the UPR is mediated by IRE-1α, which is activated by autophosphorylation and oligomerization upon accumulation of misfolded proteins in the ER. IRE-1α contains an endoribonuclease (RNase) and a kinase domain. So far, the most described function of the RNase domain is to reduce ER load through unconventional splicing of the X-box binding protein 1 (XBP1) mRNA [18]. This unconventional splicing leads to removal of a 26-nucleotide intron. Recently, it was uncovered that a multimeric protein complex tRNA splicing ligase is responsible for ligation of IRE-1α-cleaved XBP1 mRNA 5′ and 3′ extremities of which the RNA 2′,3′-cyclic phosphate and 5′-OH ligase (RTCB, also named HSPC117/C22orf28) seems to be the most essential subunit [19–22] and demonstrated a physiological role in plasma-cell differentiation [19]. This causes a frameshift in the XBP1 reading frame, thus generating a transcriptionally active protein (XBP1s). XBP1s controls expression of genes involved in protein folding, secretion, ERAD, and lipid synthesis [23, 24]. IRE-1α RNase activity is also involved in RNA degradation (pathway known as regulated IRE-1α-dependent decay or RIDD) [25].

The complex cellular mechanisms which lead to activation of the UPR and in-depth genetic functions in solid cancer cells have already been extensively discussed in a number of excellent review articles [26, 27, 1, 6, 2, 3, 28, 29]. Thereby, we will focus here on the novel role of the UPR network in acute leukemias.

## Role of the UPR in AML

 AML originates from a myeloid-committed hematopoietic stem cell (HSC) and is characterized by acquisition of genetic and epigenetic changes [30–35]. These cause aberrant activation of signaling pathways [36–38] contributing to AML pathogenesis and progression [39]. Mechanistically, for instance, transcription factor CCAAT/enhancer binding protein alpha (C/EBPα) [40] is frequently deregulated by genomic mutations causing maturation defects [41, 42]. Others include internal tandem duplications in the FMS-like tyrosine kinase 3 gene (FLT3-ITD) which correlates with poor outcome [43] or the balanced translocation t(15;17) (namely PML-RARα) representing the main oncogenic driver of the acute promyelocytic leukemia subset [44].

With a median age of 66 years at diagnosis and an overall survival rate for older AML patients of less than 10 %, the treatment response has not changed dramatically within three decades [45–47]. In younger patients, remission rates are more promising, while also in this population, ultimately ~50 % of the patients relapse within the first 5 years [38, 39, 45–50].

### UPR and *C*/*EBPα* in AML

Studies showed that the IRE-1α/XBP1s branch of the UPR was activated in 17.4 % of AML cases [51, 52]. Besides the expression of XBP1s (16 out of 92 patients), equally that of GRP78 and the ER protein quality control lectin calreticulin were increased [52]. Characteristically, these patients were poor risk (44 % vs 25 %), although this was not significant, potentially because of the low patient numbers. The important role of calreticulin was then shown by the same research group, providing evidence that it was able to bind and block the translation of C/EBPα [53]. Here, calreticulin did bind to the *C*/*EBPα* mRNA and form a stem-loop secondary structure preventing translation. Similarly, the UPR-related molecule disulfide isomerase protein (PDI), a thiol-disulfide oxidoreductase residing in the ER lumen, did equally bind to this stem-loop region of the *C*/*EBPα* mRNA [54]. Together, they formed a complex and regulated the translation of *C*/*EBPα*. Still, this observation remains surprising as both calreticulin and PDI are localized in the lumen of the ER and are not predicted to be present in the same compartment as *C*/*EBPα* mRNA.

### UPR and *PML-RARα* in AML

In the majority of PML-RARα^+^ AML patients, retinoic acid receptor alpha (RARα) is fused to the promyelocytic leukemia (PML) open reading frame on chromosome 15 [55]. The resulting fusion molecule PML-RARα acts as transcriptional repressor in a dominant-negative manner by blocking retinoic acid-induced myeloid differentiation [56]. In PML-RARα^+^ AML, only one study described a potential therapeutic role for the UPR. Mechanistically, wild-type RARα forms heterodimers with the soluble nuclear receptor co-repressor 1 (N-CoR) family of co-repressors mediating transcriptional repression [57, 58] and releases the co-repressors in response to cognate agonists such as all-trans-retinoic acid (ATRA) leading to myeloid differentiation [56]. Additionally, the N-CoR protein is critical for transcriptional repression by the tumor suppressor Max dimerization protein 1 (MAD) [59, 60].

**Fig. 1:**
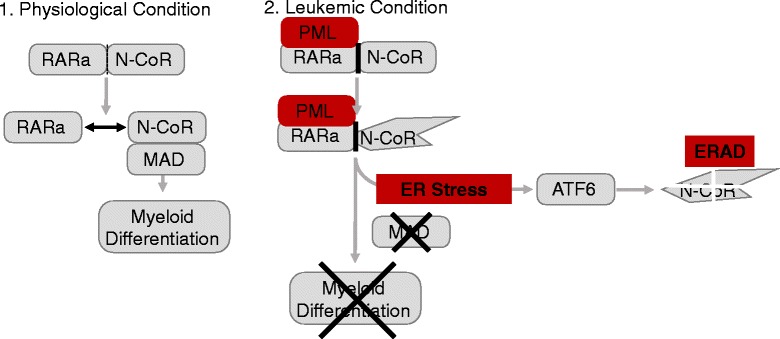
A schematic overview on how PML-RARα causes ER stress in AML is provided. Here, under physiological conditions, RARα can dissociate from coreceptors such as N-CoR. In PML-RARα^+^ AML, the binding is significantly increased. This leads to a conformational change of the N-CoR protein. The misfolded N-CoR protein is degraded by ERAD through activation of ATF6α. Through the lack of soluble and functional N-CoR, protein myeloid differentiation is prevented and activity of the tumor suppressor MAD is reduced

 Here, a mechanism is through increased binding to N-CoR by PML-RARα [60]. This binding causes an abnormal protein conformation and insolubility of the N-CoR protein. The misfolded N-CoR protein is then recruited to the ER and targeted by the ERAD system. Thereby, the tumor-suppressive function of MAD is missing. This suggests that the UPR is a critical promoter of aberrant activation of PML-RARα through ubiquitination of N-CoR [61] (Fig. 1).

### UPR and hypoxia in AML

Hypoxic environments such as the bone marrow, where the majority of leukemia cells reside [62], lead to activation of the hypoxia-inducible factor (HIF) pathway. HIF is a heterodimer consisting of an unstable alpha subunit (such as HIF-1α) and a stable beta subunit (HIF-1β) binding DNA through hypoxia-response elements. Under normoxic conditions, HIF members are hydroxylated and thereby inactivated by prolyl hydroxylase domain family members generating a binding site for the von Hippel-Lindau (pVHL) tumor suppressor. This leads to ubiquitination of HIF-α when oxygen is available. Under hypoxic conditions through the functional lack of pVHL, HIF-α accumulates and dimerizes with HIF-β family members activating several hundred genes [63].

PML-RARα has been shown to transcriptionally activate HIF-1α, while this was not through direct physical interaction. Furthermore, downregulation of HIF-1α by shRNA negatively affected self-renewal, migration, and neo-angiogenesis of PML-RARα^+^ AML cells. Additionally, inhibition of HIF-1α together with ATRA was synergistic [64]. The correlation between expression of HIF factors and the prognosis of AML patients is divergent. For instance, HIF-1α negatively impacted survival of normal karyotype (NK) AML patients, where low HIF-1α expression was associated with improved event-free survival (*p* = 0.04, hazard ratio = 0.22). Multivariate analysis showed that HIF-1α expression was an independent prognostic marker [65]. A very recent study showed an opposite result, where HIF-2α expression was not associated with poor prognosis. HIF-2α expression was even higher in good risk subgroups such as inv(16) (*p* = 0.0031) and t(15;17) (*p* = 0.04) [66, 67].

 Hypoxic conditions are well-described stress inducers leading to activation of the UPR [68]. The link between hypoxia and UPR in AML was suggested by the description that HIF-2α protected AML cells against ER stress [69]. Genetic silencing of HIF-2α did not only affect survival of long-term repopulating HSCs, but also caused an increase of ER stress and production of reactive oxygen species (ROS). The increased production of ROS and UPR led to apoptosis in AML cells [69].

In PML-RARα^+^ AML cell lines, such as HL60, the link between oxidative stress and UPR was shown as activation of the NADPH oxidase by phorbol-12-myristate-13-acetate (PMA) led to increased ROS production and caused ER stress as shown by expression and phosphorylation of different UPR genes including GRP78, eIF2α, and XBP1s [70].

## Role of the UPR in B-ALL

 In contrast to AML, ALL originates from either the B-cell (B-ALL) or T-cell lineage (T-ALL). B-ALL is the most common type of childhood leukemia and is frequently found in adults. Here, other B-ALL specific genetic alterations such as fusion genes, e.g., translocation between breakpoint cluster region and tyrosine kinase abelson murine leukemia viral oncogene homolog 1 (BCR-ABL1) [71], activating point mutations such as NRAS^G12D^, rearrangements in the mixed lineage leukemia gene (MLLr) [72], and aberrations in fundamental genes of B-cell development, e.g., paired box 5 (PAX5) and ikaros family zinc finger protein 1 (*IKZF1*) [73, 74], define B-ALL progression as well as subsequent relapse [75, 76, 74, 77].

 Mutations in the RAS/RAF pathway have been identified in ~20–30 % of B-ALL patients correlating with poor prognosis [78]. Interestingly, the recent identification that RAS mutations are significantly increased in relapsed B-ALL patients further underlines their critical clinical importance [79]. Therapeutically, activating RAS mutations such as NRAS^G12D^ is very challenging as they confer resistance towards chemotherapy [80]. More importantly, a pivotal disadvantage of targeted tyrosine kinase inhibitor (TKI) therapy, such as imatinib is paradoxical activation (off-target) of the RAS pathway [81].

### UPR and *BCR-ABL1* in B-ALL

The Philadelphia chromosome (Ph) is the result of a reciprocal translocation of BCR on chromosome 22 (region q11) and ABL1 on chromosome 9 (region q34) resulting in the constitutive active tyrosine kinase BCR-ABL1 [82]. The BCR-ABL1 fusion gene defines a high-risk subset (Ph^+^ ALL) correlating with very poor survival rates [83].

 Initial studies showed that both XBP1s and GRP78 were higher expressed in Ph^+^ leukemia cell lines, while detailed functional studies were missing [84]. Additionally, withdrawal of imatinib in Ph^+^ leukemia caused hy peractivation of the BCR-ABL1 kinase leading to metabolic reprogramming accommodated with an increase of ATP production through glycolysis. This led to increased ER swelling and stress and subsequent activation of XBP1s and CCAAT/enhancer-binding protein homologous protein (CHOP) [85], a known inducer of UPR-related apoptosis [86].

We showed that for instance, GRP78, IRE-1α, and XBP1 were upregulated in the high-risk Ph^+^ and MLLr^+^ B-ALL subsets [87]. Additionally, their promoters were also hypomethylated in B-ALL cases. Furthermore, genetic deletion of *Grp78* or *Xbp1* caused apoptosis in mouse models of BCR-ABL1 und NRAS-mutated B-ALL. Mechanistically, expression of XBP1s was linked to BCR-ABL1 kinase activity. Both genetic and pharmacological inhibition of BCR-ABL1 downstream signaling such as signal transducer and activator of transcription 5 (STAT5), mitogen-activated protein kinase/extracellular signal-regulated kinase (MAPK/ERK), or protein kinase B alpha (AKT) led to reduction of XBP1s expression. BTB and CNC homology 1, basic leucine zipper transcription factor 2 (BACH2), a tumor suppressor in B-ALL [88], was also able to negatively regulate expression of XBP1s [87].

 In the Ph^+^ ALL subsets, TKIs such as imatinib or dasatinib directly targeting the BCR-ABL fusion oncogene are essential for a successful therapy [89, 73, 83, 90]. In Ph^+^ ALL primary cases, imatinib treatment downregulated XBP1s expression, while not entirely abolishing it [87]. This suggests that a potential dual strategy targeting both the IRE-1α/XBP1 axis with a TKI could be beneficial.

Clinically, high mRNA expression of XBP1 correlated with poor prognosis [87], both in univariate and multivariate analysis in B-ALL, even in the Ph^+^ ALL subset.

## UPR and stem cells

**Fig. 2:**
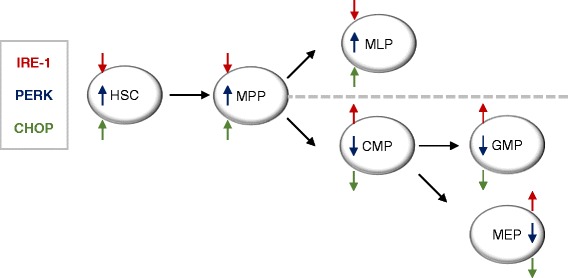
The expression profile of different UPR genes is shown by microarray analysis in HSC and different progenitor populations (multipotent progenitors (MPP), common myeloid progenitors (CMP), multipotent lymphoid progenitors (MLP), megakaryocytic-erythroid progenitors (MEP) and granulocyte-monocyte progenitors (GMP). The hierarchical tree is based on van Galen et al.

In the physiological stem cell hierarchy, the UPR has been shown to regulate the self-renewal capacities of HSCs. Through single cell analysis, it was possible to study exact expression levels in individual progenitor populations [91]. Gene expression analysis revealed that in HSCs, PERK was predominately activated compared to downstream progenitor populations (Fig. 2) [92].

 Further experiments showed that HSCs were sensitive to induced ER stress, for instance through ER stressing agents such as tunicamycin. HSCs were equally more sensitive towards ER-induced apoptosis, when compared to progenitor populations, which was mainly mediated by activation of the PERK-eIF2α-ATF4-CHOP pathway. A pharmacological PERK inhibitor (GSK2606414) caused apoptosis in the HSC population, while progenitor populations were resistant, even at high concentrations [92].

While these studies were conducted in physiological HSC populations, they raise the question, if similarly individual UPR genes play an important role in leukemia stem/initiating cells (LSCs/LICs). LICs share common characteristics of normal HSCs such as self-renewal, pluripotency, and quiescence [93] and are considered a fundamental source of relapse [94–96]. Considering the probability of trans-differentiation of cancerous stem cells into non-stem cell state and vice versa, they are usually not affected by conventional and even targeted therapies [94]. Nevertheless, thus far, an extensive study addressing whether leukemia stem cells are dependent on either global activation of the UPR or individual branches of the UPR is still missing. This could have direct therapeutic implications, since small-molecule inhibitors which target both PERK and IRE-1α signaling are currently available [87, 97–102].

## The UPR as a druggable target

### Concept of proteotoxicity as a therapeutic approach

**Fig. 3:**
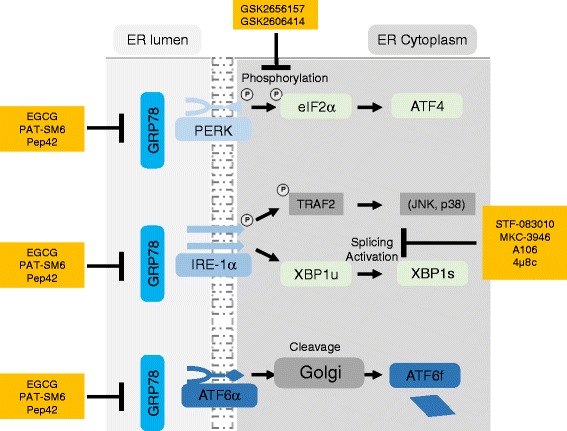
A schematic overview of the UPR network and the currently available inhibitors targeting the individual signaling molecules and subsequent pathways

The concept of proteotoxicity was mainly studied in secretory cells such as plasma cells, which as antibody-producing cells are strongly dependent on a well-developed secretory system and are pruned to potential protein overload. Here, proteasomal degradation represents the main pathway for ERAD [103]. Thereby, most studies focused on a better understanding, how plasma-cell derived malignancies, namely multiple myeloma, could be targeted through the proteasome. This led to development of the proteasome inhibitor bortezomib which blocks the 20S proteasome with great clinical success [104]. Despite great initial clinical success, extensive use caused multiple mechanisms of resistance [105] including point mutations, such as a point mutation (G322A) in the β-subunit of the binding pocket of bortezomib [106]. Another mechanism of resistance is activation of alternative degradation pathways including the aggresome [107], upregulation of heat shock proteins, or dedifferentiation of multiple myeloma cells to become less dependent on an effective ER stress control [108]. These developments have to be considered, despite the fact that disrupting proteostasis is a potential novel approach in acute leukemias (Fig. 3).

### Targeting PERK

As the UPR has already been suggested as a potential oncogenic network, the therapeutic efforts to date have focused mainly on the PERK/eIF2α and IRE-1α/XBP1 signaling axes. In the case of PERK, selective ATP-competitive *PERK* kinase inhibitors such as GSK2606414 or GSK2656157 [97, 98] were anti-proliferative in multiple cancer models in vivo including multiple myeloma. So far, pharmacological inhibition of PERK kinase activity has not yet been shown to be effective in leukemia cells. Still, a potential hint in this direction is that loss of PERK kinase activity using dominant-negative mutants already showed to negatively affect leukemia survival, albeit in chronic myeloid leukemia, but not acute leukemia cells [109].

### Targeting GRP78

Targeting GRP78 has been achieved by epigallocatechin gallate (EGCG), a green tea extract [110] which binds to the ATP-binding site of GRP78. This binding modulates its ATPase activity leading to conformational conversion into the inactive oligometric form. In B-ALL cells, treatment with EGCG caused apoptosis [111]. Additionally, targeting GRP78 also sensitized B-ALL cells towards vincristine, a chemotherapeutic drug commonly used in the therapy of B-ALL patients.

In a second approach, Pep42, a cyclic 13-mer peptide [112], was used to target both cell surface and intracellular GRP78 after receptor-mediated endocytosis and was equally able to induce apoptosis in B-ALL cell lines [111]. So far, two clinical studies are studying GRP78 as a therapeutic target. EGCG is currently being evaluated in Alzheimer’s disease (clinical trial ID: NCT00951834), while a novel monoclonal IgM antibody (PAT-SM6) against GRP78 [113, 114] is being evaluated in multiple myeloma with moderate clinical response (clinical trial ID: NCT01727778) [115]. Single treatment with PAT-SM6 led to a stable disease in four out of 12 patients (Table 1).

**Table 1:** Overview of clinical trials investigating the therapeutic usefulness of the UPR

Compound	Molecular target	Disease	Stage of clinical development	References
Epigallocatechin gallate (EGCG)	GRP78	Alzheimer’s disease	Clinical, phase 2 and 3	NCT00951834
PAT-SM6	GRP78	Multiple myeloma	Clinical, phase 1	NCT01727778, Rasche et al. [115]

### Targeting IRE-1α/XBP1s signaling

 Most currently available small-molecule inhibitors block the RNase activity either through direct inhibition of the RNase [101, 102] or through modulation of the kinase domain [99]. The currently available preclinical small-molecule inhibitors (MKC-3946, STF-083010, A-I06 and 4μ8c) aim to prevent *XBP1* mRNA splicing and production of the transcriptionally active XBP1s protein [87, 97–102, 116]. In the concept of using proteotoxicity as a therapeutic approach, most initial studies focused on multiple myeloma with only partially convincing results. For instance, treatment with MKC-3946 led to apoptosis in multiple myeloma cells [101]; nevertheless, this is in contrast to other studies which suggest that multiple myeloma cells can survive without functional XBP1s [108].

In B-ALL, we tested the efficacy of IRE-1α inhibition and were able to show that treatment with different preclinical IRE-1α inhibitors caused apoptosis in primary B-ALL cases in a dose-dependent manner [87]. XBP1s splicing was significantly reduced in primary B-ALL cases, and this was accommodated with ER stress as shown by increased accumulation of a specific ER-tracker [117]. Pharmacological inhibition of IRE-1α was able to cause not only apoptosis, but also cell cycle arrest in B-ALL xenografts and prolonged survival of B-ALL-bearing mice in vivo [87].

 Nevertheless, small-molecule inhibitors for IRE-1α seem to vary substantially in their efficacy and specificity [99, 116, 100, 101]. The identification of a new class of hydroxy-aryl-aldehydes of IRE-1α inhibitors [118] might help to improve our understanding of small-molecule inhibitor design [119], allowing improved inhibition of IRE-1α RNase activity.

 Additionally, the identification of the RIDD mechan ism, through which IRE-1α can splice mRNAs containing a specific consensus sequence which is recognized by the RNase domain [120], suggests that genetic and pharmacological results may vary, depending for instance on the type of cancer, or lead to activation of other secondary pathways.For instance, pharmacological inhibition of the RNase domain of IRE-1α predominantly blocked splicing of XBP1, while RIDD remained intact [121]. This suggests that splicing of RIDD targets and XBP1 differs substantially, and both should be considered as distinct pharmacological targets.

A final aspect is the acquisition of secondary mutations or resistance, a major therapeutic challenge for instance in TKI-treatment. Until now, no mutations for XBP1 have been identified yet in B-ALL patients, suggesting that this pathway is active. Still, the identification of two loss-of-function mutations of XBP1 in multiple myeloma [108] suggests that the use of IRE-1α inhibitors could lead to acquisition of mutations in acute leukemias [122, 108] (Table 2).

**Table 2:** Overview of preclinical studies studying the therapeutic usefulness of the UPR

Compound	Molecular target	Disease	Stage of clinical development	References
STF-083010	IRE-1	Multiple myeloma	Preclinical	Papandreou et al. [99]
		ALL		Kharabi Masouleh et al. [87]
		CLL		Kriss et al. [116]
A106	IRE-1	CLL	Preclinical	Kriss et al. [116]
		ALL		Kharabi Masouleh et al. [87]
MKC-3946	IRE-1	Multiple myeloma	Preclinical	Volkmann et al. [100]
				Mimura et al. [101]
4μ8c	IRE-1	Multiple myeloma	Preclinical	Cross et al. [102]
GSK2606414	PERK	Multiple myeloma	Preclinical	Axten et al. [98]
GSK2656157		Pancreatic cancer		
Epigallocatechin gallate (EGCG)	GRP78	ALL	Preclinical	Uckun et al. [111]
PAT-SM6	GRP78	Melanoma	Preclinical	Rosenez et al. [113]
		Multiple myeloma		Rasche et al. [114]
Pep42	GRP78	ALL	Preclinical	Uckun et al. [111]

## Concluding remarks

 Taken together, the role of the UPR in acute leukemia subsets has only recently begun to be elucidated, while further studies are required to answer several questions and comprehend the global impact of UPR signals in such disease. Several studies indicate that different UPR genes play an important role in both AML and B-ALL. Still, both diseases are quite heterogeneous, and it has to be elucidated how different oncogenes cause activatio of UPR. For instance, BCR-ABL1 kinase signaling significantly differs to that of MLLr. Still, in both B-ALL subsets, XBP1s expression was highly upregulated [87]. Additionally, the impact of the UPR on LSCs is unknown. The study by van Galen et al. suggests that different progenitor populations show distinct expression and dependencies on the UPR [92]. Translating these findings to LSCs suggests that LSCs might not be dependent equally on all UPR pathway, rather only on individual pathways, while such a study has not yet been conducted.

Finally, the finding that expression of XBP1s is significantly reduced but not abolished upon treatment with different pharmacological inhibitors (MEK, AKT, or BCR-ABL) suggests that potential combinational therapies with IRE-1α inhibitors might be useful [87].
